# *Porphyromonas gingivalis* Peptidylarginine Deiminase, a Key Contributor in the Pathogenesis of Experimental Periodontal Disease and Experimental Arthritis

**DOI:** 10.1371/journal.pone.0100838

**Published:** 2014-06-24

**Authors:** Neville Gully, Richard Bright, Victor Marino, Ceilidh Marchant, Melissa Cantley, David Haynes, Catherine Butler, Stuart Dashper, Eric Reynolds, Mark Bartold

**Affiliations:** 1 Colgate Australian Clinical Dental Research, School of Dentistry, University of Adelaide, Adelaide, South Australia, Australia; 2 Discipline of Anatomy and Pathology, School of Medical Sciences, University of Adelaide, Adelaide, South Australia, Australia; 3 Oral Health Collaborative Research Centre, Melbourne Dental School, The University of Melbourne, Melbourne, Victoria, Australia; University of Florida, United States of America

## Abstract

**Objectives:**

To investigate the suggested role of *Porphyromonas gingivalis* peptidylarginine deiminase (PAD) in the relationship between the aetiology of periodontal disease and experimentally induced arthritis and the possible association between these two conditions.

**Methods:**

A genetically modified PAD-deficient strain of *P. gingivalis* W50 was produced. The effect of this strain, compared to the wild type, in an established murine model for experimental periodontitis and experimental arthritis was assessed. Experimental periodontitis was induced following oral inoculation with the PAD-deficient and wild type strains of *P. gingivalis*. Experimental arthritis was induced via the collagen antibody induction process and was monitored by assessment of paw swelling and micro-CT analysis of the radio-carpal joints. Experimental periodontitis was monitored by micro CT scans of the mandible and histological assessment of the periodontal tissues around the mandibular molars. Serum levels of anti-citrullinated protein antibodies (ACPA) and *P. gingivalis* were assessed by ELISA.

**Results:**

The development of experimental periodontitis was significantly reduced in the presence of the PAD-deficient *P. gingivalis* strain. When experimental arthritis was induced in the presence of the PAD-deficient strain there was less paw swelling, less erosive bone damage to the joints and reduced serum ACPA levels when compared to the wild type *P. gingivalis* inoculated group.

**Conclusion:**

This study has demonstrated that a PAD-deficient strain of *P. gingivalis* was associated with significantly reduced periodontal inflammation. In addition the extent of experimental arthritis was significantly reduced in animals exposed to prior induction of periodontal disease through oral inoculation of the PAD-deficient strain versus the wild type. This adds further evidence to the potential role for *P. gingivalis* and its PAD in the pathogenesis of periodontitis and exacerbation of arthritis. Further studies are now needed to elucidate the mechanisms which drive these processes.

## Introduction

The endogenous microbes inhabiting humans often interact in complex ways with their hosts. Changes in the local environment can lead to qualitative and/or quantitative changes in commensal microbial communities that, if left unchecked, can result in disease. Chronic periodontitis is a common inflammatory condition affecting the tissues surrounding teeth. A prolonged, uncontrolled inflammatory response to the sub-gingival microbial load may lead to loss of periodontal ligament attachment and the adjacent alveolar bone [1]. In recent years periodontitis has been linked to the development of other disorders, such as coronary heart disease, diabetes mellitus and low birth weight [2]. While these associations are largely based on epidemiological evidence and for most there is currently no apparent common underlying cause, dysregulation of the inflammatory response seems to be a common underlying feature [3]. Two of the most prevalent chronic inflammatory conditions affecting humans that share many common features, including destruction of both fibrous connective tissue and bone, osteoclast activation and many common risk factors, are periodontitis and rheumatoid arthritis (RA) [4], [5].

While elevated microbial load is an important factor in the initiation of periodontitis, it is the increase in proportion of specific microbial pathogens that is likely to be the crucial factor in the subsequent progression of this condition [6]. Periodontitis, particularly in its more severe form, has been linked to a biofilm that contains a consortium of oral pathogens that includes the Gram negative anaerobe *Porphyromonas gingivalis*. [7], [8]. *P. gingivalis* expresses a peptidylarginine deiminase (PAD) known as PPAD, an enzyme that modifies peptidylarginine residues to citrulline and is unique in this regard amongst prokaryotes [9]. PPAD is not evolutionarily related to the mammalian PADs that catalyse the same reaction, which is the modification of the guanidino group of arginine residues to produce peptidyl-citrulline and ammonia. While citrullination mediated by host PADs is generally considered a fundamental process (e.g apoptosis), it is also associated with inflammation in mammals [10].

When investigating potential common causal links between periodontitis and RA, the ability of *P. gingivalis* to citrullinate peptides is noteworthy as auto-antibodies against citrullinated peptides are highly specific and sensitive in RA diagnosis [11]. Post-translationally modified peptides and proteins containing citrulline can exhibit altered epitopes compared to those that are unmodified [12]. Accordingly, citrullination has been reported to trigger an auto-immune response [11], [13] via modified self-proteins and peptides perceived as foreign by the immune system [14]. While citrullinated peptides may be involved in the pathogenesis of RA the nature of their role is unclear and the contributions of host or prokaryote PADs to citrullination is unknown.

It has been proposed that the increased levels of *P. gingivalis* in patients suffering from chronic periodontitis might influence the development of RA, via PPAD promotion of peptide citrullination, thus explaining the over representation of patients presenting with periodontitis suffering from RA [15]–[17].

Therefore the aims of this investigation were to construct a PAD-deficient *P. gingivalis* strain and compare the onset and severity of arthritis and periodontitis in a mouse model in the presence of either the wild type or PAD-negative strain.

## Materials and Methods

### Ethics

Approval for the use of BALB/c mice in this study was obtained from the University of Adelaide, Animal Ethics Committee (Project N° M-2012-183R). The animals were housed in the University of Adelaide PC2 Animal holding facility (OGTR certification No 2067/2008). Approval to culture and prepare inoculates of the genetically modified *P. gingivalis* strain ECR527 was granted by the Institutional Biosafety Committee of the University of Adelaide (Approval No: IBC Dealing ID 10890). The animals were assessed daily for a number of general health parameters including dull/ruffled coat, a change in temperament, reduced food/water intake or a reluctance to move and body weight recorded.

### Bacterial Strains and Culture Conditions

*P. gingivalis* strain W50 (wild type) was obtained from the culture collection of the Oral Health CRC, Melbourne Dental School, University of Melbourne. A PAD-deficient strain (ECR527) was derived from W50 for this study. *P. gingivalis* strains were routinely maintained on Horse Blood Agar (HBA) plates and antibiotic selection of 10 µg/mL erythromycin when appropriate. *E. coli* alpha-select gold cells (Bioline, London, UK) were grown at 37°C in Luria Bertani (LB) Broth or maintained on LB agar with 100 µg/mL ampicillin when harbouring pGEM-TEasy plasmids (Promega, Madison, WI, USA).

### DNA Analysis and Manipulations

Oligonucleotide primers used in this study are listed in Table 1. Genomic DNA from *P. gingivalis* strains was prepared using the DNeasy Blood and Tissue kit (Qiagen) and plasmid DNA was extracted from *E. coli* using the QIAprep spin miniprep kit (Qiagen, Venlo, Netherlands). DNA was sequenced by Applied Genetics Diagnostics, The University of Melbourne.

**Table 1:** Oligonucleotide primers used in this study.

Oligonucleotide Primer	Sequence (5′-3′)^a^	Purpose
PG1424-RT-Fwd	ATCGAAGCAGATGTCGTCTCAT	RT PCR
PG1423-RT-Rev	TACGAAACCAATGCTCAGATTTTG	RT PCR
PG1422-RT-Rev	ATAGTTGGATGCGAGAGAAGGA	RT PCR
PG1426-Fwd	GAAGCACGTAATAAGGACAATGAC	*pad* recombination cassette
ErmF-PG1426-Rev	ACGGGCAATTTCTTTTTTGTCAT TGTTTGATATGTTTTATGATGTTATGAA	*pad* recombination cassette
PG1426-ErmF-Fwd	TTCATAACATCATAAAACATATCAAACA ATGACAAAAAAGAAATTGCCCGT	*pad* recombination cassette
PG1423-ErmF-Rev	GTATTCTCAAATAAGGGGCC TTACGAAGGATGAAATTTTTCAGG	*pad* recombination cassette
ErmF-PG1423-Fwd	CCTGAAAAATTTCATCCTTCGTAA GGCCCCTTATTTGAGAATAC	*pad* recombination cassette
PG1423-Rev	CATCGACGATGATATCTCCTGT	*pad* recombination cassette

a Underlined sequence of SOE primers indicates the part of the primer that is not complementary to the target sequence, but provides complementarity with a second PCR product for splicing.

### RT-PCR

RNA was isolated using a NucleoSpin RNA II Total RNA Isolation kit (Macherey-Nagel, Duren, Germany) and cDNA was generated using a SuperScrip III Reverse Transcriptase First-Strand Synthesis SuperMix for qRT-PCR kit with random hexamers (Life Technologies, California, USA), both according to the manufacturer’s instructions. BIOTAQ Red DNA Polymerase (Bioline, London, UK) was then used to PCR from the following templates: 125 ng of cDNA, 125 ng RNA that was not reverse transcribed, milliQ H_2_O and 13 ng W50 genomic DNA in 50 µL reactions with gene-specific primer pairs (Table 1) for 30 cycles in a G-Storm GS1 thermal cycler (Gene Works, Adelaide, Australia). The expected product size from each primer pair was 682 bp for PG1424-RT-Fwd and PG1423-RT-Rev; and 1181 bp from PG1424-RT-Fwd and PG1422-RT-Rev. DNA was electrophoresed at 80 V for 40 min using a 1.0% agarose gel prepared with tris-acetate buffer (40 mM tris-acetate, 1 mM EDTA, pH 8).

### Construction of *P. gingivalis* PAD-deficient Strain ECR527

The recombination cassette for deletion of the *pad* gene consisted of 972 bp upstream of the *PG1424* (*pad*) ORF which encompassed *PG1426*, followed by the *ermF* gene encoding erythromycin resistance in *P. gingivalis*, followed by 741 bp downstream of the *PG1424* ORF which encompassed *PG1423*. This recombination cassette resulted in replacement of *PG1424* with *ermF* and was constructed from three separate PCR products that were spliced together using gene splicing by overlap extension PCRs (SOE PCR) to form the final cassette. SOE PCRs were performed essentially as previously described [18]. The PCR products that flanked the PG1424 ORF were amplified from W50 genomic DNA: *PG1426* (primers PG1426-Fwd and ErmF-PG1426-Rev) and *PG1423* (primers ErmF-PG1423-Fwd and PG1423-Rev). The *ermF* gene was amplified from pVA2198 [19] with primers PG1426-ErmF-Fwd and PG1423-ErmF-Rev. All PCRs were performed with Herculase II DNA Polymerase (Stratagene, La Jolla, Ca, USA) except for the final SOE PCR which was amplified with Platinum Taq DNA Polymerase High Fidelity (Life Technologies, California, USA). PCR products were purified using the NucleoSpin Extract II purification kit (Macherey Nagel, Duren, Germany) according to manufacturer’s instructions. The final PCR product was ligated with pGEM-TEasy and transformed into *E. coli* alpha-select gold competent cells (Bioline) by heat shock according to manufacturer’s instructions. The resulting plasmid was sequenced to confirm the fidelity of the recombination cassette then it was linearised and 200 ng electroporated into *P. gingivalis* W50 in a 0.1 cm gap cuvette at 1.8 kV, 200 Ohms resistance. The resulting PAD-deficient strain was named ECR527.

### Measurement of PAD Activity

A colorimetric microtitre plate assay for the determination of enzymatic deimination of the substrate N-α-benzoyl-L-arginine ethyl ester (BAEE) was performed as previously described [20], [21]. Briefly, cells from three biological replicate cultures each of *P. gingivalis* W50 and ECR527 (OD_650_ 1.1–1.3) were washed and resuspended in 0.2 M Tris-HCl (pH 8), 1 mM EDTA, 1 µM flavin mononucleotide and 10 mM cysteine. A 10 µL aliquot of BAEE (30 mM) was added to 50 µL cells (1×10^8^ cfu) and incubated at 37°C for 30 min before addition of 200 µL of detection reagent. The detection reagent was assembled daily from its two components with one part A (0.5% diacetyl monoximine and 0.01% thiosemicarbazide) added to two parts B (0.25 mg of FeCl_3_/mL in 24.5% sulphuric acid and 17% phosphoric acid). The reaction plate was heated at 105°C for 25 min, then cooled and optically measured at 490 nm using a Bio-Tek Powerwave spectrophotometer (Biotek Instruments, Winooski, VT, USA). A standard curve was generated using 0–400 µM L-citrulline using the same method to quantify citrulline in samples.

### Animal Groupings

Thirty six female BALB/c mice between 6 and 8 weeks old were acquired from the Laboratory Animal Services (LAS) of the University of Adelaide. Six experimental groups each containing six mice were used. All groups received oral inoculations for the duration of the study and three groups in addition to the inoculations were induced with experimental arthritis (EA) using a mouse monoclonal antibody against type II collagen (Arthrogen-CIA Arthritogenic Monoclonal Antibody, Chondrex Inc., Redwood, WA, USA).

Group 1: Control: 2% Carboxylmethylcellulose (CMC) (Sigma, St Louis, MO, USA)
Group 2: PPAD^−^: PAD-deficient *P. gingivalis* (ECR527)
Group 3: PPAD^+^: Wild type *P. gingivalis* (W50)
Group 4: Control + EA: 2% CMC with induced EA
Group 5: PPAD^−^ + EA: PAD-deficient *P. gingivalis* (ECR527) with induced EA
Group 6: PPAD^+^ + EA: Wild type *P. gingivalis* (W50) with induced EA

### Induction of Experimental Periodontitis by Oral Inoculation

The preparation of the inocula and the inoculation protocol to induce an experimental periodontitis in female BALB/c mice has been previously described [22]. All mice were inoculated with either wild type *P. gingivalis* (Groups 3 and 6) or PAD-deficient *P. gingivalis* (Groups 2 and 5). The control groups (Groups 1 and 4) were inoculated with the suspension vehicle for *P. gingivalis* strains, 2% CMC. PPAD activity for both strains was assessed prior to the preparation of each inoculum using the colorimetric method described above. Animals were inoculated over two intensive sequences, each comprising four inoculations over an eight day period. The first sequence commenced two days after the mice had completed 7 days of antibiotic treatment of 1 mg/mL kanamycin (Sigma, St. Louis, MO, USA) in deionised water *ad libitum*. The inoculation sequences were separated by a period of two weeks and during this period mice were inoculated twice a week. Following the second intensive inoculation sequence all mice continued to be inoculated twice a week for the remainder of the experimental period. All animal inoculations were performed by experienced professional staff within a Class II Biological safety cabinet and PC2 animal holding facility.

### Induction of Collagen Antibody-induced Arthritis - Experimental Arthritis

Experimental arthritis (EA) was induced in Groups 4–6 by intravenous tail vein injection with 1.5 mg of a mouse monoclonal antibody against type II collagen (Arthrogen-CIA Arthritogenic Monoclonal Antibody, Chondrex Inc., Redwood, WA, USA). This procedure was performed two days after completion of the second intensive inoculation sequence (day 44). To aid in the induction of EA a dose of 5 µg of lipopolysaccharide (LPS) was intraperitoneally administered three days after the antibody exposure, as described previously [23].

### Visual Scoring of Paw Swelling

Front and rear paws were visually assessed and scored each day by two experienced observers to assess swelling and inflammation. Two different scales were used to assess paw changes throughout the experiment. Firstly each inflamed toe or knuckle received a score of 1 and a fully inflamed swollen wrist/ankle a score of 5. Each assessed paw received a score between 0–15 and the total score per mouse was 0–60 [24]. The second system employed a scoring system whereby each paw was allocated a score from zero to four according to the level of inflammation and swelling to make a total score of 16 for each animal. 0 = normal paw, 1 = mild but definite redness and swelling of the wrist/ankle, 2 = moderate swelling and redness of the wrist/ankle with digit involvement, 3 = severe swelling and redness of the paw with digit involvement and 4 = maximum inflammation the entire paw, with multiple digit involvement.

### Live Animal Micro-computed Tomography

Mice were scanned using the Skyscan 1076 High Resolution live animal computed tomography (micro CT) (SkyScan, Bruker, Belgium) to determine changes in bone volume in the radio-carpal joint and cemento-enamel junction to the alveolar bone crest (CEJ-ABC) length in the jaws. The scanning width was set to 35 mm with a resolution of 9 µm. Detailed specifications for micro CT imaging have previously been published [22]. Animals were anaesthetised prior to scanning with a mixture of ketamine and xylazine and positioned on a polystyrene foam holder then placed within an enclosed container with a HEPA filter at the ends. The container was placed in the 3 cm carbon fibre bed of the micro CT scanner. Mice were scanned before the induction of experimental inflammatory disease to obtain baseline measurements and again at the completion of the study (10 days post experimental arthritis induction).

### Micro-CT Data Processing

Scans were reconstructed using Skyscan NRECON software (Version 1.6.6.0). Settings used; smoothing = 1, ring artefact = 15, beam hardening = 30% and misalignment compensation was adjusted manually using the DataViewer program (Skyscan, Bruker, Belgium). The BMP files created were opened in CT Analyser (Version 1.12.0.0+) to create a volume of interest. To realign images and save the appropriate plane for either the radio-carpal joint or determining the CEJ-ABC distance, DataViewer (Version 1.4.4 64-bit) was used. Transaxial images were saved for the bone volume determinations in the radio-carpal joint, and sagittal images for the CEJ-ABC distance. The images were then opened in CTAnalyser to measure bone volume or CEJ-ABC distance. The histogram settings were set at 100 and 255 to measure both the bone volume in the radio-carpal joint and CEJ-ABC junction distance. The bone volume was calculated in a set number (200) 9 µm slices above and below a standard reference point set within the radiocarpal joint. The CEJ-ABC distance was measured between the second and third molars on three slices for each mouse [22], [25].

### Serum Collection

Approximately 100 µL of blood was collected from cheek puncture bleeds at the commencement of the study (Time = 0) and at 6 weeks. Cardiac puncture was used at the conclusion of the study (week 9). Blood was allowed to clot for 1 hour at room temperature, centrifuged at 1000 *g* for 20 min at room temperature and the serum removed and dispensed into aliquots and stored prior to analysis at −80°C.

### ELISA Assays

An anti-CCP kit (AXIS-Shield, Dundee, Scotland) was modified and used to assess levels of anti-CCP antibodies in mouse sera. Week 9 serum was diluted 1∶200 and analysed according to the manufacturer’s instructions by substitution of an anti-mouse horseradish peroxidase-conjugated secondary to detect mouse IgG (Cell Signalling Technology, Boston, MA, USA).

Antibodies to wild type *P. gingivalis* were detected by coating Costar Maxisorp 96 well plates (Corning, Tewksbury, MA, USA) with 1×10^8^ formalin fixed cells in 50 mM bicarbonate buffer, pH 9.4, overnight at 4°C. Wells were washed 3 times in PBS/0.05% Tween-20 (PBS-T) and blocked with 5% skim milk in PBS-T for 1 hr at room temperature followed by three washes of PBS-T. An antibody against *P. gingivalis* purchased from the Developmental Studies Hybridoma Bank (Iowa City, IA, USA) was used as a control and to produce a standard curve. Mouse serum samples from time (day 0, 6 weeks and 9 weeks) were diluted 1∶100, added to wells in duplicate and incubated at room temperature for 2 hrs. Wells were washed three times and incubated with a mouse horseradish peroxidase-conjugated secondary antibody (1∶10000) (Cell Signalling Technology, Boston, MA, USA) for 45 minutes at room temperature. After the final washes the substrate reagent was prepared (R&D, Minneapolis, MN, USA) and added to wells. The colour reaction was stopped with 2 M H_2_SO_4_ and the plate read at 450 nm on the Power-wave plate reader using dedicated KC4 microplate data analysis software (Biotek Instruments, Winooski, VT, USA).

### Histological Evaluation

At the conclusion of the study (day 63) mice were euthanized by CO_2_ inhalation. All paws and heads were retrieved from each mouse and placed into 10% buffered formalin for two days. Following fixation, the specimens were rinsed thoroughly in physiological buffered saline (PBS) and then decalcified in 10% EDTA for 14 days with regular replacement of the solution over this period. Complete decalcification was confirmed by digital radiography and specimens paraffin embedded and sectioned at a thickness of 7 µm. Sections were stained with haematoxylin and eosin for histological analysis. Slides were viewed using a Leica DM1000 microscope and imaged with a connected Leica DFC450 Camera system (Leica Microsystems, Wetzlar, Germany). The front and rear paws were assessed for inflammatory changes, bone and cartilage destruction and pannus formation. A point scale (0–3) was used to score the severity of inflammatory changes based on the number and type of inflammatory cells, (0 = normal tissue, 0–5% inflammatory cells; 1 = mild inflammation 5–25% inflammatory cells; 2 = moderate inflammation 25–50% inflammatory cells; 3 = severe inflammation >50% inflammatory cells). Bone and cartilage destruction was assessed in a similar fashion (0 = normal, 1 = mild cartilage destruction, 2 = evidence of bone and cartilage destruction, 3 = severe bone and cartilage destruction. Pannus formation in the joint was also noted. The periodontium of the maxillary molars was assessed for inflammation with interdental papilla reduction, and presence of osteoclasts with bone erosion particularly between the first and second molars. The severity of each parameter was scored separately on a scale from 0 to 3 (0 = normal, 1 = mild effect, 2 = moderate effect, 3 = severe effect). The sections assessed and selected for each mouse were all at the level where the distal root of the first molar and proximal root of the second molar was clearly visible [23].

### Recovery of *P. gingivalis* from the Oral Cavity

Three groups of six mice were inoculated with either 2% CMC (vehicle control), wild type *P. gingivalis* or PAD-deficient *P. gingivalis*. The inoculation sequence employed was identical to that of Groups 1–3 described previously. At 0, 6 and 24 hrs after the final inoculation sequence the animals gingivae were swabbed using periodontal strips and these were used to inoculate Brain Heart Infusion Broth. Broth cultures were gown under anaerobic conditions for seven days and an inoculum from each culture spread onto anaerobic blood agar plates. Following seven days of anaerobic incubation a typical black-pigmented colony from each group was Gram stained and assayed for PPAD activity.

### Statistical Analysis

The data collected from all surviving mice in this study was analysed by one-way ANOVA. When the global test was statistically significant (p<0.05), post hoc comparisons were performed using the Tukey or Bonferroni’s Multiple Comparison, 95% confidence level. Unpaired t-tests were also used for 2 group comparisons. All testing was carried out with Graph Pad Prism 6 as the statistical analysis package. Significance levels were set at p<0.05 for all tests.

## Results

### Construction of PAD-deficient *P. gingivalis* Strain (ECR527)

Peptidylarginine deiminase is encoded on the wild type *P. gingivalis* genome at locus PG1424. It is predicted by MicrobesOnline [26] to be the first gene in a 3 gene operon with PG1423 and PG1422, which we have confirmed in *P. gingivalis* W50 using reverse transcription PCR. The RT-PCR products corresponded to the predicted sizes of 682 bp for the primer pair specific for PG1424/PG1423 and 1181 bp for the primer pair specific for PG1424/PG1422 (data not shown). A recombination cassette was constructed using gene splicing by Overlap Extension PCRs where the open reading frame of the *ermF* gene, which confers erythromycin resistance was inserted in place of the PG1424 ORF, so that *ermF* would be expressed from the PG1424 promoter. Replacement of the PG1424 ORF with the *ermF* ORF was successfully achieved in *P. gingivalis* strain W50 with the resulting mutant (ECR527) noticeably slower to pigment than wild type W50. Loss of PPAD activity by the PAD-deficient *P. gingivalis* was confirmed biologically by loss of the ability of ECR527 cultures to deiminate the substrate BAEE whereas 1×10^8^ cfu of wild type *P. gingivalis* produced 20.4±0.8 nmol N-α-benzoylcitrulline ethyl ester in 30 min from 300 nmol BAEE.

### Post Inoculation Recovery of *P. gingivalis* from Gingiva

Following incubation, black-pigmented colonies of Gram negative bacteria were observed on blood agar plates from the swabs obtained at 0, 6 and 24 hours post final inoculation with wild type *P. gingivalis* W50 and PAD-deficient *P. gingivalis*. However no growth was observed from swabs of animals inoculated with 2% CMC vehicle control. PPAD activity was positive only for bacteria recovered from animals exposed to W50 (Table 2).

**Table 2:** Recovery of viable *P. gingivalis* from oral swabs after final inoculation.

Group	Recovery 0 hr	Recovery 6 hr	Recovery 24 hr	PPAD activity
Control	−	−	−	−
Wild-type (W50)	+	+	+	*
PAD-deficient (ECR527)	+	+	+	−

(+) Viable wild type (W50) and PAD-deficient (ECR527) *P. gingivalis strains* W50 and ECR527 were recovered from the gingiva of each group of mice inoculated separately with each strain at the three time points assayed. The colonies in both groups were black pigmented with gram negative staining organisms.

(−) No viable organisms were recovered from the control group receiving the vehicle (2% CMC).

(*) PPAD activity was only demonstrated in the group of mice where *P. gingivalis* W50 was recovered from gingival swabs.

### Micro-CT Analysis of Periodontal Bone Loss

**Figure 1:**
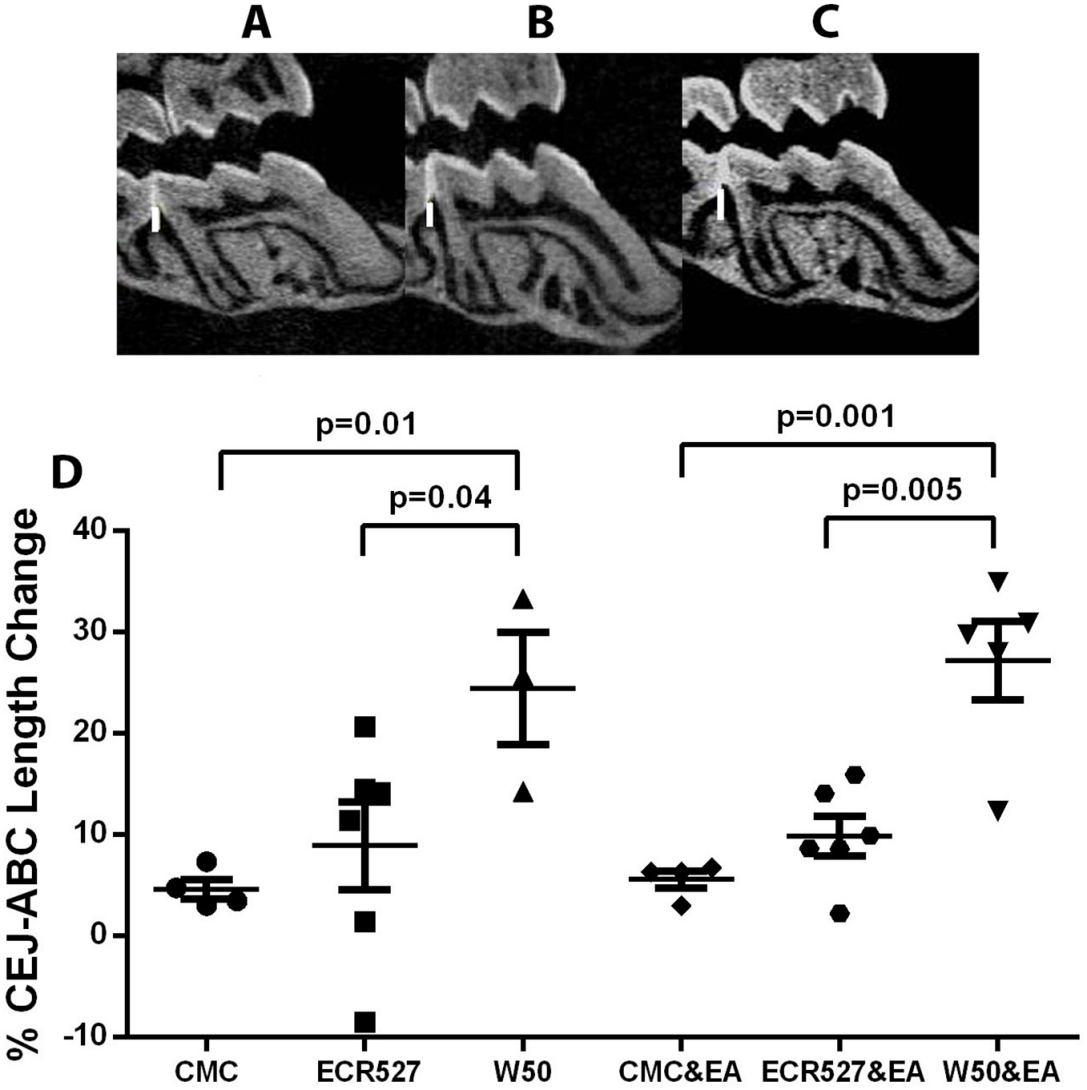
Micro CT appearance and analysis of periodontal bone loss. A. Micro CT of mouse jaw for CMC control group. B. Micro CT of mouse jaw for ECR527 & EA group. C. Micro CT of mouse jaw W50 & EA group. D. Measurements of bone changes in the jaw were calculated by measuring the cemento-enamel junction-alveolar bone crest on three slices for each mouse using CTAn sagital sections. Data represent mean (± SEM). The number of animals in each group was 6 and measurements were performed in triplicate. Statistical analysis was done by one way ANOVA and Tukey multiple comparisons. White bars represent CEJ/ABC distance measurements. *Abbreviations:* CMC = carboxymethyl cellulose; ECR527 = PAD-deficient *P. gingivalis*; W50 = wild type *P. Gingivalis*; EA = experimental arthritis

To determine the effect of PPAD on the periodontium, bone loss in the jaws of the mice, the cemento-enamel junction to alveolar bone crest (CEJ-ABC) length was measured in all 6 groups (Figure 1). No significant difference between PAD-deficient *P. gingivalis* and the CMC vehicle control was observed. However a significant increase in CEJ-ABC distance reflecting increased bone loss was observed between wild type P. *gingivalis* and CMC vehicle control (p = 0.01) and wild type *P. gingivalis* with experimental arthritis and CMC vehicle control with experimental arthritis (p = 0.001). Furthermore, there was significantly higher bone loss between wild type *P. gingivalis* W50 and PAD-deficient *P. gingivalis* (p = 0.04) over wild type *P. gingivalis* with experimental arthritis and PAD-deficient *P. gingivalis* with experimental arthritis (p = 0.005).

### Histological Analysis of Maxillary Periodontal Tissues

**Figure 2:**
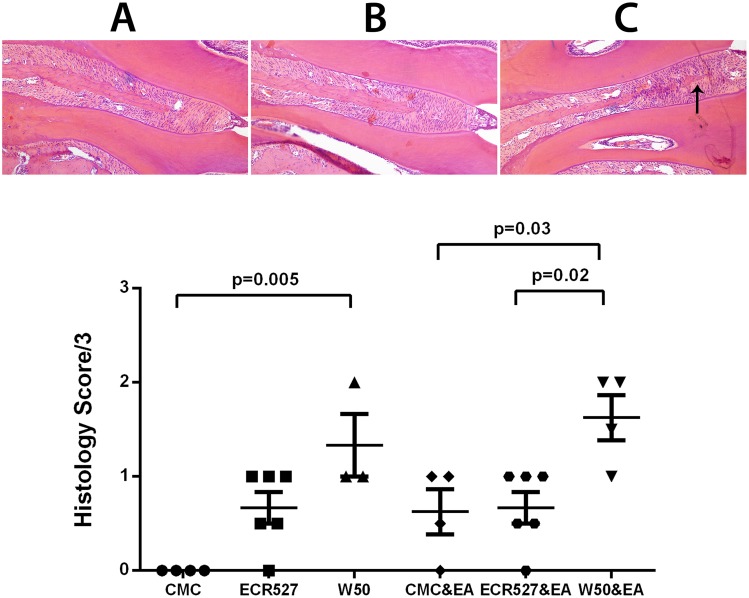
Histological analysis of maxillary periodontal tissues. A. Histology of mouse jaw for CMC control group at day 63. B. Histology of mouse jaw for ECR527 & EA group at day 63. C. Histology of mouse jaw for W50 & EA group at day 63. Arrow indicates increased inflammatory reaction in supra-crestal alveolar bone gingival tissue. D. Histological scores (mean ± SEM) for the presence of osteoclasts and evidence of bone erosion around the first and second upper molars for all groups in the study. Original magnification of A–C = 100X. The number of animals in each group was 6 and measurements were performed in triplicate. Statistical analysis was done by one way ANOVA and Tukey multiple comparisons. *Abbreviations:* CMC = carboxymethyl cellulose; ECR527 = PAD-deficient *P. gingivalis*; W50 = wild type *P. Gingivalis*; EA = experimental arthritis.

Histological assessment of the periodontal tissue around the first and second maxillary molars demonstrated a statistically significant number of osteoclasts and evidence of bone resorption in the experimental arthritis groups where the difference between the PAD-deficient *P. gingivalis* and the CMC groups were also significantly different to the wild type *P. gingivalis* group (p = 0.03 and p = 0.02 respectively; Figure 2). There was no statistical difference seen between the non-experimental arthritis groups and the experimental arthritis group which had CMC or PAD-deficient *P. gingivalis* inoculations. Interestingly, the control group with induced experimental arthritis showed evidence of bone resorption which was not seen in the CMC group where there was no induced experimental arthritis.

### Micro-CT Analysis of Bone Erosion in Front Paw Radio-carpal Joint

**Figure 3:**
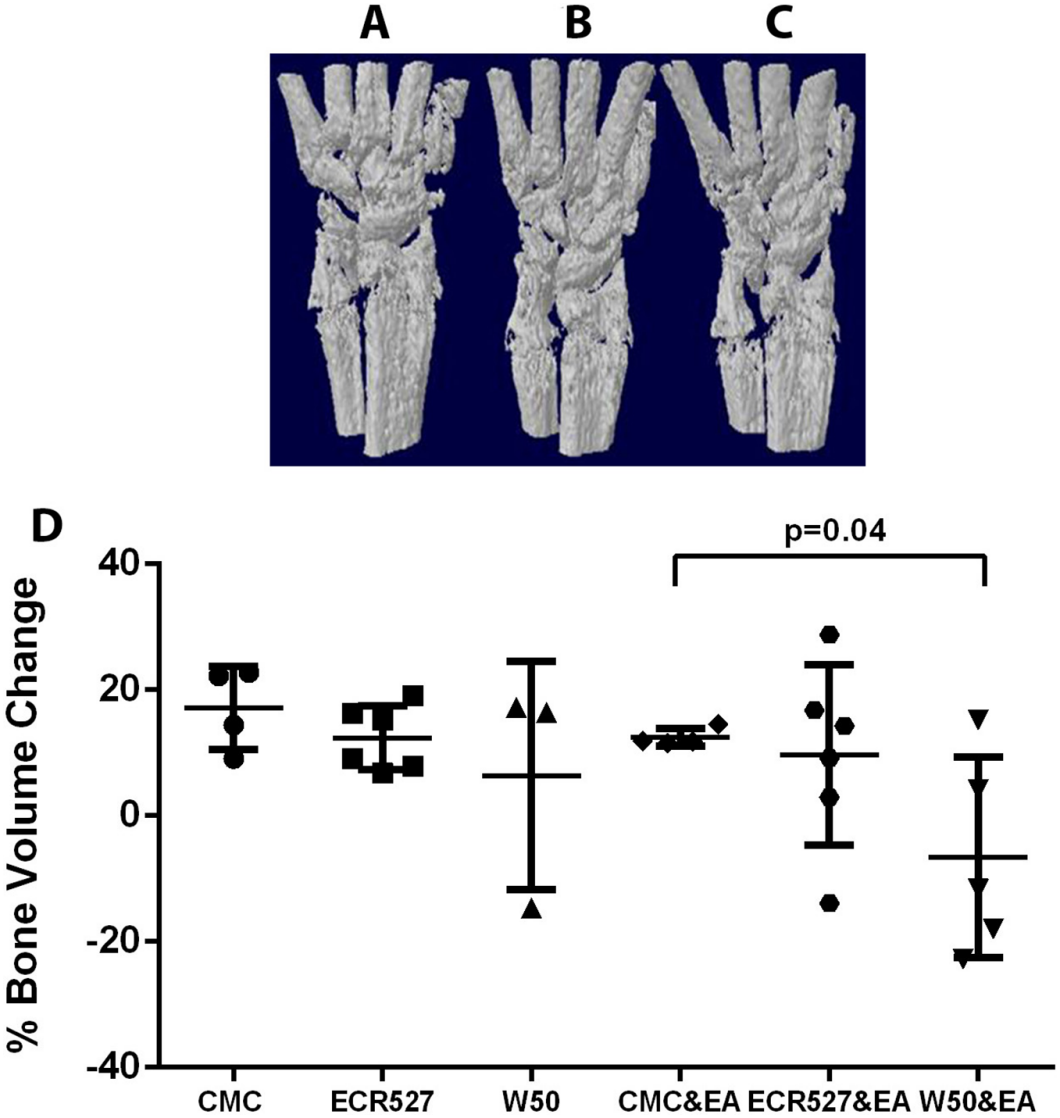
Micro CT appearance and analysis of bone erosion in front paw radio-carpal joint. A. Micro CT appearance of paw for CMC control group at day 63. B. Micro CT appearance of paw for ECR527 & EA group at day 63. C. Micro CT appearance of paw for W50 & EA group at day 63. D. The front paws (left and right combined), were processed to determine bone loss/growth between the two scanned time points in the radio-carpal joint. Data represent mean (± SEM). The number of animals in each group was 6 and measurements were performed in duplicate. Statistical analysis was done by one way ANOVA and Bonferroni multiple comparisons. *Abbreviations:* CMC = carboxymethyl cellulose; ECR527 = PAD-deficient *P. gingivalis*; W50 = wild type *P. Gingivalis*; EA = experimental arthritis

Animals were micro-CTscanned and the radio-carpal joints of both paws (combined) were analysed for changes in bone volume over time. The W50 group with EA showed significantly lower bone volume change reflecting increased bone loss, than the CMC with experimental arthritis group in the radio-carpal joint (p<0.04) (Figure 3). Other group comparisons were not significant.

### Visual Scoring of Paw Swelling

**Figure 4:**
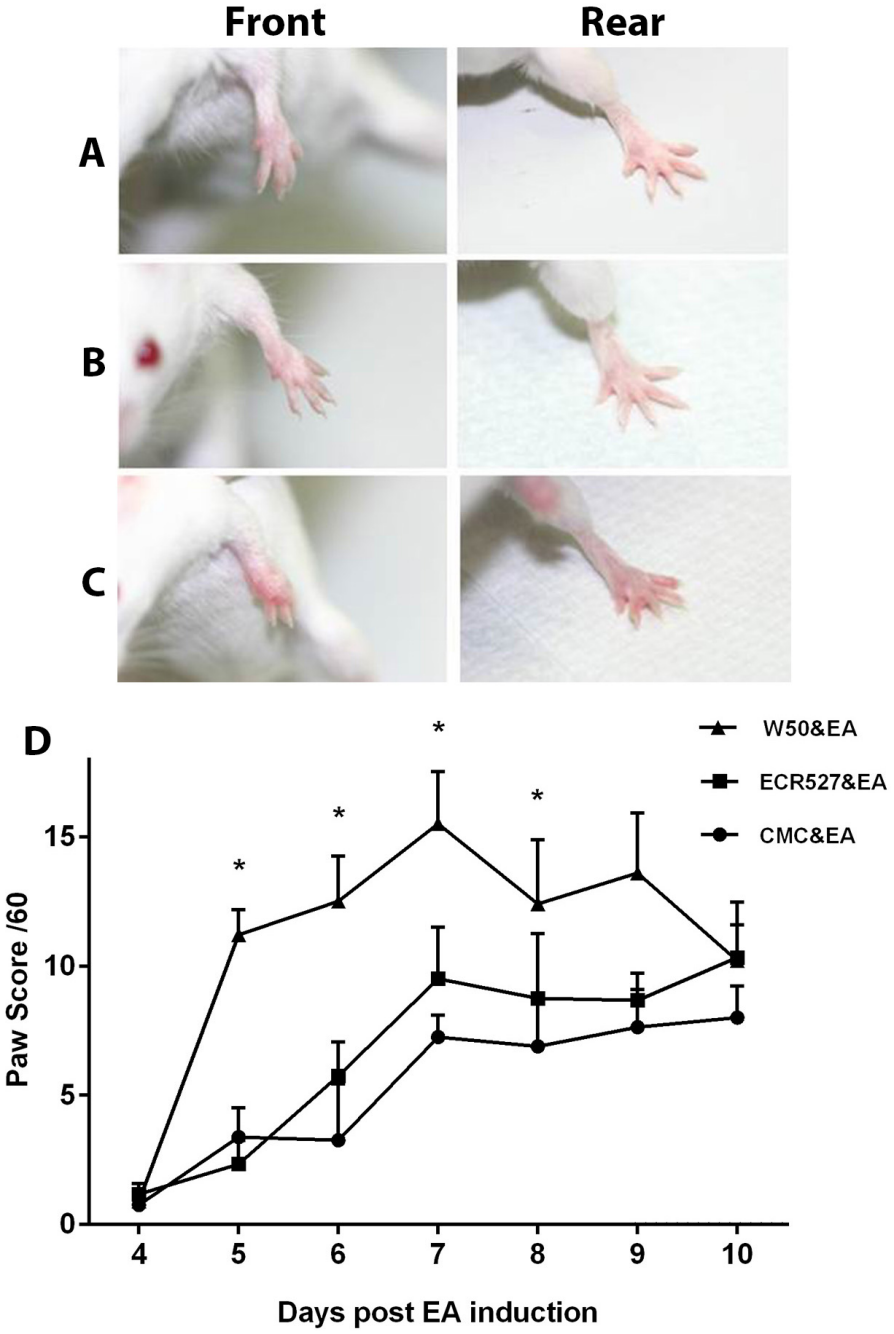
Visual appearance and scoring of paw swelling. A. Appearance of front and rear paws for CMC control group at day 7 post EA induction. B. Appearance of front and rear paws for ECR527 & EA group at day 7 post EA induction. C. Appearance of front and rear paws for W50 & EA group at day 7 post EA induction. D. Total paw score grade out of a 60 including front and back paws. Data represent mean (± SEM). The number of animals in each group was 6 and measurements were performed once daily per animal Statistical analysis was done by two way ANOVA and Tukey multiple comparisons. Raw data for data points are shown in Figure S1. *Abbreviations:* CMC = carboxymethyl cellulose; ECR527 = PAD-deficient *P. gingivalis*; W50 = wild type *P. Gingivalis*; EA = experimental arthritis

Front and rear paws of all mice induced with experimental arthritis were scored daily for swelling and inflammation. There was no significant difference between the control (CMC inoculated group) and the group inoculated with PAD-deficient *P. gingivalis* for the onset of disease and maximum paw score at the height of the disease (day 6–7). However this was not the case for the group that received wild type *P. gingivalis*. There was a rapid onset with swelling and inflammation evident at day 4 and continued for the next 3 days with paw scores significantly higher (p<0.0001) than those of the CMC and PAD-deficient *P. gingivalis* groups (Figure 4).

### Histological Assessment of Joint Inflammation

**Figure 5:**
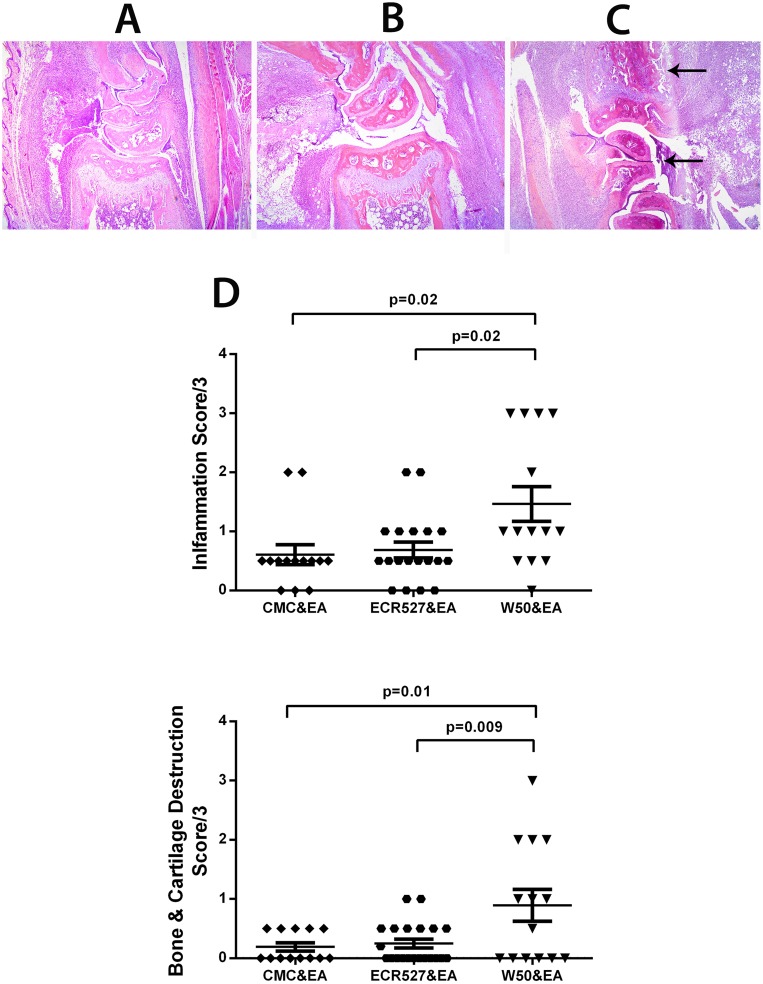
Histological assessment of joint inflammation. A. Histology of the radio-carpal joint for CMC control group at day 63. B. Histology of the radio-carpal joint for ECR527 & EA group at day 63. C. Histology of the radio-carpal joint for W50 & EA group at day 63. Arows indicate increased inflamaotry reaction in joint. D. Histological scores (mean ± SEM) for (a) inflammation and (b) bone and cartilage destruction for both front and rear paws combined. Original magnification of A–C = 100X. The number of animals in each group was 6 and measurements were performed in triplicate. Statistical analysis was done by One way ANOVA and Bonferroni multiple comparisons. *Abbreviations:* CMC = carboxymethyl cellulose; ECR527 = PAD-deficient *P. gingivalis*; W50 = wild type *P. Gingivalis*; EA = experimental arthritis

Histological assessment of the sections of inflamed paws in the experimental arthritis group of animals confirmed the observations from the clinical paw scores that the level of inflammation and cartilage and bone destruction was significantly higher in the group inoculated with wild type control groups (p = 0.02 and 0.01 respectively). A significant difference was also noted between the wild type *P. gingivalis* and PAD-deficient strain for inflammation and bone/cartilage destruction (p = 0.02 and p = 0.009 respectively). No differences were observed between the PAD-deficient strain and the CMC control groups (Figure 5). There was no paw inflammation observed in groups that did not undergo experimental arthritis induction.

### Serum anti-CCP Antibody Levels

**Figure 6:**
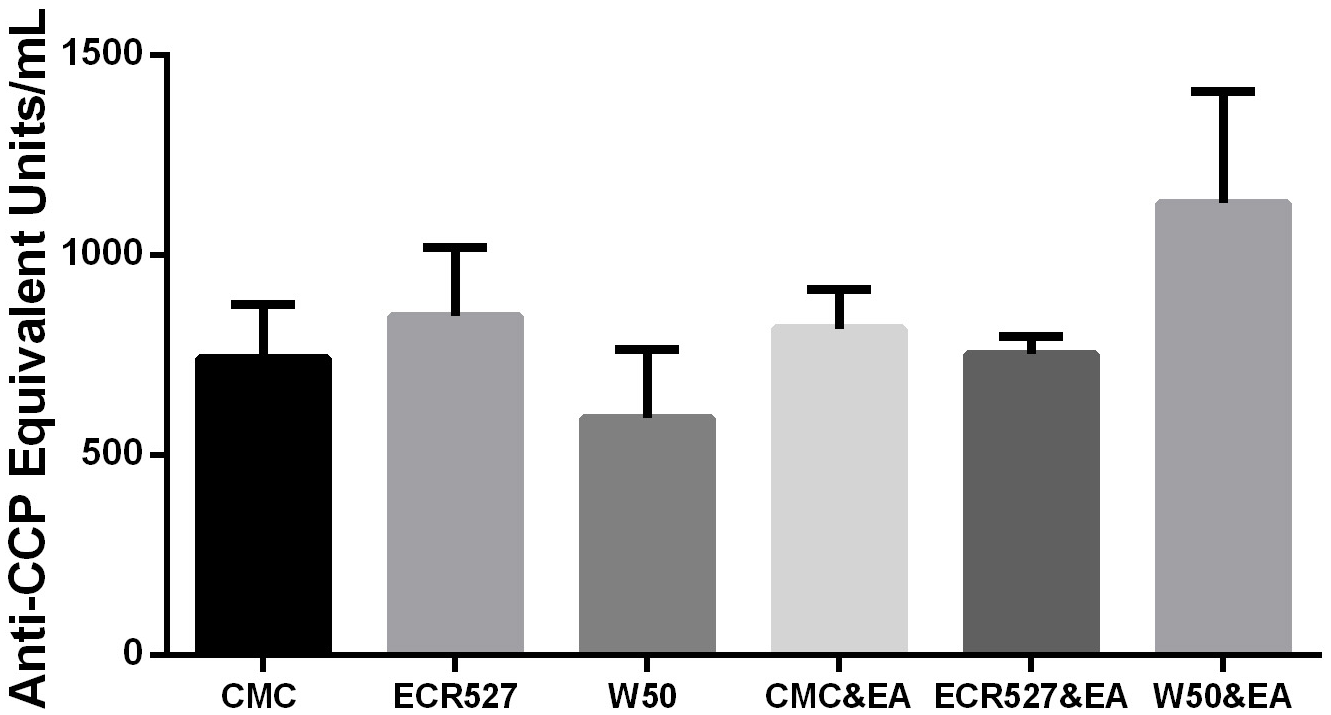
Serum anti-CCP antibody levels. The mean (± SEM) titre of anti-CCP antibodies detected in the sera of mice at week 9 from each of the 6 treatment groups. Raw data for data points are shown in Figure S2. The number in each group was 6 and measurements were performed in duplicate. Statistical analysis was done by one way ANOVA and Tukey multiple comparisons. *Abbreviations:* CMC = carboxymethyl cellulose; ECR527 = PAD-deficient *P. gingivalis*; W50 = wild type *P. Gingivalis*; EA = experimental arthritis

Control mice (inoculated with CMC) demonstrated a background level of anti-CCP antibodies (ACPA). Similar levels were demonstrated in the groups inoculated with wild type *P. gingivalis* and PAD-deficient *P. gingivalis* strains. There was no significant increases in anti-CCP titre in the CMC and *P. gingivalis* ECR527 inoculated groups compared to the groups with no EA. Only mice induced with EA and with pre-existing periodontitis induced by *P. gingivalis* W50 demonstrated a trend towards an increase in anti-CCP antibodies, but this was not significant (Figure 6).

### Serum anti-*P. gingivalis* Levels

**Figure 7:**
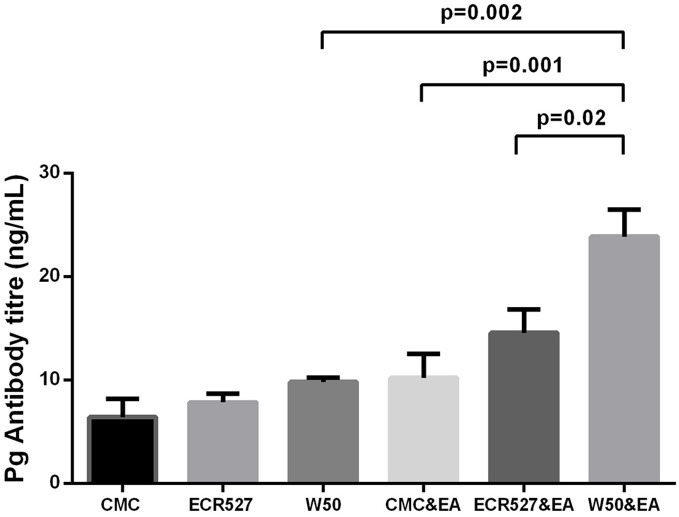
Serum anti-*P. gingivalis* levels. The mean (± SEM) titre of anti-*P.gingivalis* detected in the sera of mice over 9 week time course. Raw data for data points are shown in Figure S3. The number in each group was 6 and measurements were performed in duplicate. Statistical analysis was done by one way ANOVA and Tukey multiple comparisons. *Abbreviations:* CMC = carboxymethyl cellulose; ECR527 = PAD-deficient *P. gingivalis*; W50 = wild type *P. Gingivalis*; EA = experimental arthritis

Mice developed antibodies to *P. gingivalis* over the course of the experiment. Antibody titres increased significantly following induction of experimental arthritis at week 9 in all groups. However those inoculated with wild type *P. gingivalis* produced a higher titre than experimental arthritis alone (p = 0.001) and experimental arthritis plus PAD-deficient *P. gingivalis*. (p = 0.02) (Figure 7)

## Discussion

Of the many hypotheses proposed for a linking mechanism between periodontal disease and RA, the role of citrullination and production of anti-cyclic citrullinated antibodies is of particular interest [15], [27]. Citrullination is a common post-translational modification based on the conversion of arginine into citrulline. It occurs frequently in various tissues of the body, particularly at sites of inflammation, and is initiated by peptidylarginine deiminases (PADs). A key concept in the pathogenesis of RA is the preclinical phase which precedes the clinical manifestation of RA [28]. This concept is predicated on the identification of serum autoantibodies years before the development of RA. Indeed there is good evidence to suggest that the immunological conflict often predates the onset of RA by several years. A key component in this process is the production of anti-citrullinated protein antibodies [10]. The production of citrullinated proteins and anti-citrullinated protein antibodies at extra-articular sites prior to the development of RA is well documented [10]. It has been proposed that the inflamed periodontal tissues may be one such site [29].

A number of animal models of experimental arthritis have been studied, each presenting certain advantages and disadvantages [30]. The model chosen for this study, collagen antibody induced arthritis, is characterized by macrophage and polymorphonuclear leukocyte infiltrate within the joints. In this model the anti-collagen antibody is adoptively transferred and therefore it does not rely on specific B- and T-cell responses for disease to ensue. Nonetheless the development of arthritis in the CAIA model is similar to other models with regards to the inflammatory cytokine response and pannus formation within the affected joints [31], [32].

While the role of anti-citrullinated protein antibodies in the pathogenesis of rheumatoid arthritis has been controversial, there is good evidence to support a pathogenic role for these antibodies in the development of both experimental arthritis and human rheumatoid arthritis. [33], [34]. While not studied in as much detail as other experimental arthritis models, anti-citrullinated protein antibodies have been suggested to play a role in the severity of disease in the collagen antibody induced arthritis model of experimental arthritis [35], [36]. To date there have been no reports concerning an anti-citrullinated protein antibody response in experimental periodontitis. In this regard the present study is significant since it reports that mice with experimental periodontitis and subsequent induction of experimental arthritis demonstrated a trend (not significant) towards an increase in anti-citrullinated protein antibody titre. The precise role that anti-citrullinated protein antibodies play in this process await further investigation.

Shortly after *P. gingivalis* was shown to be the only prokaryote to produce a peptidylarginine deiminase (PPAD) a hypothesis was presented in which the ability of this organism to citrullinate proteins through secretion of PPAD could be one mechanism whereby anti-citrullinated protein antibodies could develop and result in an exaggerated antibody response with subsequent joint inflammation and development of rheumatoid arthritis [9], [15].

To explore this concept further we constructed a knock-out *P. gingivalis* strain deficient in PPAD expression (ECR527) with view to examining the role of PPAD in the development of both experimental periodontitis and arthritis. We have recently completed sequencing of the PAD-deficient *P. gingivalis* on our in-house ion torrent and found no secondary mutations, indicating that the experimental results were solely due to the deletion of *pad* from *P. gingivalis* (unpublished data).

There is only one other report in the literature that has used a PAD-deficient *P. gingivalis* strain in a experimental arthritis model [37]. However, our study complements and extends this earlier study [37] by investigating experimental periodontitis in addition to experimental arthritis and a combination of both. This is an important extension of the earlier study [37] which investigated the effect of subcutaneous inoculation of *P. gingivalis* on experimental arthritis only. Furthermore we demonstrated that both the wild type and PAD-deficient strain of *P. gingivalis* survived in the oral cavity after oral inoculation. Recent observations indicate that experimental periodontitis can only progress in the presence of a biofilm containing viable *P. gingivalis* [38]. In this study we used *P. gingivalis* W50 as it is highly virulent in murine periodontitis models [39]–[45].

With regards to the induction of experimental periodontitis it was noteworthy that oral inoculation with the PAD-deficient strain resulted in a significantly reduced amount of periodontal bone loss compared to oral inoculation with the wild type *P. gingivalis*. The reasons for this response are as yet unclear and require further investigation but this finding does implicate a role for PAD in the pathogenesis of periodontitis.

When periodontitis was first induced followed by induction of experimental arthritis studies it was noted that inoculation with the PAD-deficient *P. gingivalis* strain resulted in reduced severity and onset of arthritis. This is in agreement with a previous study in which it was noted that inoculation with PAD-deficient strain of *P. gingivalis* resulted in reduced severity of collagen-induced experimental arthritis [37].

In recent years the concepts of molecular mimicry and posttranslational modification of proteins in auto-immune reactions such as RA have been actively debated [28], [46]. Interestingly, *P. gingivalis* appears as a likely agent involved in these processes. For example, cross reactivity of antibodies to *P. gingivalis* heat shock protein (HSP 60) and host-derived heat shock protein in the pathogenesis of RA has been documented [47]. Another case for molecular mimicry involving *P. gingivalis* has been made whereby antibodies against citrullinated human α-enolase cross react with citrullinated *P. gingivalis* enolase [27]. Similarly, the ability of *P. gingivalis* to produce PPAD which can citrullinate host proteins and lead to an early anti-citrullinated protein antibody response has also implicated this bacterium in the pathogenesis of RA [37]. While antibodies to PPAD have been detected in the sera of mice injected with *P. gingivalis*, the titre is very low and this reaction probably does not contribute to the process of *P. gingivalis*-induced exacerbation of experimental arthritis [37]. In the present study, when periodontitis was induced the anti-citrullinated protein antibody serum levels for control and PAD-deficient strains were similar. However, following induction of experimental arthritis the anti-citrullinated protein antibody titres were elevated only for the wild type strain and not for the controls or PAD-deficient strains. This implies a synergistic role for PPAD in the development of experimental arthritis. This observation is in line with those from a study using a different model of collagen-induced experimental arthritis [37].

The emerging data now significantly implicate local citrullination within inflamed periodontal tissues as having the potential to be an extra-articular source of citrullination and production of anti-citrullinated protein antibodies. To date, smoking has been considered a major risk factor in the pathogenesis of RA whereby smoking leads to citrullination within the lung and a priming anti-citrullinated protein antibody response which leads to exacerbation of the anti-citrullinated protein antibody response and associated tissue destruction within inflamed joints. In light of recent observations of the presence of inflammation-associated citrullination, presence of PAD-2 and PAD-4 in gingival tissues [28], [48] as well as PPAD in serum [49] now provides good evidence for another important extra-articular source for citrullination and anti-citrullinated protein antibody production to occur years in advance of the development of the clinical signs and symptoms of RA. In this regard it is significant that individuals with untreated RA who are non-smokers but presenting with chronic periodontitis have a significantly high level of anti-citrullinated protein antibody titres [50].

In conclusion this study has demonstrated that PPAD appears to be a significant factor in the development of experimental periodontitis and exacerbation of experimental arthritis. Interpretation of these findings must be made in the context that the animal models used do not completely reflect the complexities of human dises for both periodontitis and rheumatoid arthrits.

Nonetheless these findings adds further evidence for a potentail role of *P. gingivalis* in the pathogenesis and exacerbation of arthritis. Further studies are now needed to elucidate the mechanisms which drive these processes.

## Supporting Information

Figure S1 **Raw data used for determination of data points in** **Figure 4** **.**
(DOCX)

Figure S2 **Raw data used for determination of data points in** **Figure 6** **.**
(DOCX)

Figure S3 **Raw data used for determination of data points in** **Figure 7** **.**
(DOCX)

Data S1 (PDF)
