# Supplementary material for: Porphyromonas gingivalis Peptidylarginine Deiminase, a Key Contributor in the Pathogenesis of Experimental Periodontal Disease and Experimental Arthritis
Source: PLoS One. 2014 Jun 24;9(6):e100838. doi: 10.1371/journal.pone.0100838 (PMC4069180; doi:10.1371/journal.pone.0100838)
Supplement: Figure S1 — Raw data used for determination of data points in Figure 4 . (DOCX) [file pone.0100838.s001.docx]

**Figure S1**

**Paw Score/60**

|  |  | **Days** | **4** | **5** | **6** | **7** | **8** | **9** | **10** |
| --- | --- | --- | --- | --- | --- | --- | --- | --- | --- |
|  | **M2** |  | 1 | 2 | 0 | 7 | 3 | 4 | 5 |
|  | **M3** |  | 1 | 6 | 0.5 | 5 | 8 | 7 | 8 |
| **CMC&EA M4** | |  | 1 | 4.5 | 9.5 | 9 | 11 | 11 | 11 |
|  | **M5** |  | 0 | 1 | 3 | 8 | 5.5 | 8.5 | 8 |
|  |  | **Mean** | **0.75** | **3.38** | **3.25** | **7.25** | **6.88** | **7.63** | **8** |
|  |  | **SD** | **0.5** | **2.29** | **4.37** | **1.71** | **3.42** | **2.93** | **2.45** |
|  |  | **SEM** | **0.25** | **1.14** | **2.18** | **0.85** | **1.71** | **1.46** | **1.22** |
|  | **M1** |  | 0.5 | 6.5 | 10.5 | 5 | 20 | 13 | 18 |
|  | **M2** |  | 0.5 | 0.5 | 3.5 | 17 | 6 | 9 | 7 |
|  | **M3** |  | 1 | 1.5 | 9 | 8 | 5 | 7 | 8 |
|  | **M4** |  | 1.5 | 2 | 4.5 | 14 | 9 | 10 | 15 |
| **ECR&EA M5** | |  | 0.5 | 0.5 | 3 | 5 | 2.5 | 6 | 4 |
|  | **M6** |  | 3 | 3 | 4 | 8 | 10 | 7 | 10 |
|  |  | **Mean** | **1.17** | **2.33** | **5.75** | **9.5** | **8.75** | **8.67** | **10.33** |
|  |  | **SD** | **0.98** | **2.25** | **3.17** | **4.93** | **6.15** | **2.58** | **5.24** |
|  |  | **SEM** | 0.4 | 0.92 | 1.3 | 2.01 | 2.51 | 1.05 | 2.14 |
|  | **M2** |  | 1 | 14 | 17.5 | 21 | 21 | 20 | 14 |
|  | **M3** |  | 1.5 | 11 | 16 | 17 | 15 | 18 | 13 |
|  | **M4** |  | 0 | 8 | 9 | 15 | 8 | 10 | 8 |
| **W50&EA M5** | |  | 1 | 11 | 10 | 16 | 9 | 12 | 7 |
|  | **M6** |  | 1 | 12 | 10 | 8.5 | 9 | 8 | 9 |
|  |  | **Mean** | **0.9** | **11.2** | **12.5** | **15.5** | **12.4** | **13.6** | **10.2** |
|  |  | **SD** | **0.55** | **2.17** | **3.94** | **4.53** | **5.55** | **5.18** | **3.11** |
|  |  | **SEM** | **0.24** | **0.97** | **1.76** | **2.02** | **2.48** | **2.32** | **1.39** |

**Figure S1. Raw data used for determination of data points in Figure 4.**
