# Supplementary material for: Porphyromonas gingivalis Peptidylarginine Deiminase, a Key Contributor in the Pathogenesis of Experimental Periodontal Disease and Experimental Arthritis
Source: PLoS One. 2014 Jun 24;9(6):e100838. doi: 10.1371/journal.pone.0100838 (PMC4069180; doi:10.1371/journal.pone.0100838)
Supplement: Figure S2 — Raw data used for determination of data points in Figure 6 . (DOCX) [file pone.0100838.s002.docx]

**Figure S2**

**Anti-CCP antibody titre**

|  | **CMC** | **ECR527** | **W50** | **CMC&EA** | **ECR527&EA** | **W50&EA** |
| --- | --- | --- | --- | --- | --- | --- |
|  | 584.41 | 1405.37 | 344.28 | 966.211 | 787.48 | 698.5 |
|  | 584.41 | 1327.3 | 382.17 | 991.835 | 863.95 | 647.76 |
|  | 635.08 | 1366.3 | 1043.16 | 559.097 | 762.04 | 2296.65 |
|  | 1392.34 | 1483.72 | 889.48 | 546.445 | 762.04 | 2242.37 |
|  | 749.32 | 559.1 | 445.32 | 812.951 | 622.41 | 711.2 |
|  | 736.61 | 521.15 | 457.95 | 685.809 | 546.45 | 673.12 |
|  | 647.76 | 521.15 |  | 927.819 | 673.12 | 1171.91 |
|  | 609.74 | 508.51 |  | 1043.156 | 711.2 | 1107.45 |
|  |  | 445.32 |  |  | 749.32 | 889.48 |
|  |  | 407.43 |  |  | 774.76 | 863.95 |
|  |  | 851.19 |  |  | 902.25 |  |
|  |  | 774.76 |  |  | 889.48 |  |
| **Mean** | 742.46 | 847.61 | 593.73 | 816.67 | 753.71 | 1130.24 |
| **STDEV** | 270.05 | 425.03 | 295.60 | 197.56 | 105.70 | 626.47 |
| **SEM** | 37.13 | 173.52 | 30.94 | 97.61 | 43.15 | 280.17 |

**Figure S2. Raw data used for determination of data points in Figure 6.**
