# Supplementary material for: Porphyromonas gingivalis Peptidylarginine Deiminase, a Key Contributor in the Pathogenesis of Experimental Periodontal Disease and Experimental Arthritis
Source: PLoS One. 2014 Jun 24;9(6):e100838. doi: 10.1371/journal.pone.0100838 (PMC4069180; doi:10.1371/journal.pone.0100838)
Supplement: Figure S3 — Raw data used for determination of data points in Figure 7 . (DOCX) [file pone.0100838.s003.docx]

**Figure S3**

***P.gingivalis* antibody titre**

|  | **CMC** | **ECR527** | **W50** | **CMC&EA** | **ECR527&EA** | **W50&EA** |
| --- | --- | --- | --- | --- | --- | --- |
|  | 11.3 | 7.77 | 10.5 | 4.67 | 9.85 | 26.64 |
|  | 2.87 | 11.16 | 9.93 | 8.94 | 14.04 | 15.42 |
|  | 5.92 | 7.11 | 9.11 | 11.52 | 13.62 | 20.27 |
|  | 5.69 | 5.81 |  | 15.81 | 24.64 | 28.65 |
|  |  | 6.22 |  |  | 9.66 | 28.52 |
|  |  | 9.17 |  |  | 15.78 |  |
| **Mean** | 6.45 | 7.87 | 9.85 | 10.24 | 14.60 | 23.90 |
| **STDEV** | 3.52 | 2.00 | 0.70 | 4.67 | 5.49 | 5.84 |
| **SEM** | 1.76 | 0.82 | 0.40 | 2.33 | 2.24 | 2.61 |

**Figure S3. Raw data used for determination of data points in Figure 7.**
