# Supplementary material for: Porphyromonas gingivalis Peptidylarginine Deiminase, a Key Contributor in the Pathogenesis of Experimental Periodontal Disease and Experimental Arthritis
Source: PLoS One. 2014 Jun 24;9(6):e100838. doi: 10.1371/journal.pone.0100838 (PMC4069180; doi:10.1371/journal.pone.0100838)
Supplement: Data S1 — (PDF) [file pone.0100838.s004.pdf]

Figure 4 Paw Score/60

		Days	4	5	6	7	8	9	10
	**M2**		1	2	0	7	3	4	5
	**M3**		1	6	0.5	5	8	7	8
**CMC&EA**	**M4**		1	4.5	9.5	9	11	11	11
	**M5**		0	1	3	8	5.5	8.5	8
		**Mean**	**0.75**	**3.38**	**3.25**	**7.25**	**6.88**	**7.63**	**8**
		**SD**	**0.5**	**2.29**	**4.37**	**1.71**	**3.42**	**2.93**	**2.45**
		**SEM**	**0.25**	**1.14**	**2.18**	**0.85**	**1.71**	**1.46**	**1.22**
	**M1**		0.5	6.5	10.5	5	20	13	18
	**M2**		0.5	0.5	3.5	17	6	9	7
	**M3**		1	1.5	9	8	5	7	8
	**M4**		1.5	2	4.5	14	9	10	15
**ECR&EA**	**M5**		0.5	0.5	3	5	2.5	6	4
	**M6**		3	3	4	8	10	7	10
		**Mean**	**1.17**	**2.33**	**5.75**	**9.5**	**8.75**	**8.67**	**10.33**
		**SD**	**0.98**	**2.25**	**3.17**	**4.93**	**6.15**	**2.58**	**5.24**
		**SEM**	0.4	0.92	1.3	2.01	2.51	1.05	2.14
	**M2**		1	14	17.5	21	21	20	14
	**M3**		1.5	11	16	17	15	18	13
	**M4**		0	8	9	15	8	10	8
**W50&EA**	**M5**		1	11	10	16	9	12	7
	**M6**		1	12	10	8.5	9	8	9
		**Mean**	**0.9**	**11.2**	**12.5**	**15.5**	**12.4**	**13.6**	**10.2**
		**SD**	**0.55**	**2.17**	**3.94**	**4.53**	**5.55**	**5.18**	**3.11**
		**SEM**	**0.24**	**0.97**	**1.76**	**2.02**	**2.48**	**2.32**	**1.39**

Figure 6 Anti-CCP antibody titre

	CMC	ECR527	W50	CMC&EA	ECR527&EA	W50&EA
	584.41	1405.37	344.28	966.211	787.48	698.5
	584.41	1327.3	382.17	991.835	863.95	647.76
	635.08	1366.3	1043.16	559.097	762.04	2296.65
	1392.34	1483.72	889.48	546.445	762.04	2242.37
	749.32	559.1	445.32	812.951	622.41	711.2
	736.61	521.15	457.95	685.809	546.45	673.12
	647.76	521.15		927.819	673.12	1171.91
	609.74	508.51		1043.156	711.2	1107.45
		445.32			749.32	889.48
		407.43			774.76	863.95
		851.19			902.25	
		774.76			889.48	
**Mean**	742.46	847.61	593.73	816.67	753.71	1130.24
**STDEV**	270.05	425.03	295.60	197.56	105.70	626.47
**SEM**	37.13	173.52	30.94	97.61	43.15	280.17

Figure 7 *P.ging* antibody titre

	CMC	ECR527	W50	CMC&EA	ECR527&EA	W50&EA
	11.3	7.77	10.5	4.67	9.85	26.64
	2.87	11.16	9.93	8.94	14.04	15.42
	5.92	7.11	9.11	11.52	13.62	20.27
	5.69	5.81		15.81	24.64	28.65
		6.22			9.66	28.52
		9.17			15.78	
**Mean**	6.45	7.87	9.85	10.24	14.60	23.90
**STDEV**	3.52	2.00	0.70	4.67	5.49	5.84
**SEM**	1.76	0.82	0.40	2.33	2.24	2.61
